# The Crowded Crosstalk between Cancer Cells and Stromal Microenvironment in Gynecological Malignancies: Biological Pathways and Therapeutic Implication

**DOI:** 10.3390/ijms20102401

**Published:** 2019-05-15

**Authors:** Rosalba De Nola, Alessio Menga, Alessandra Castegna, Vera Loizzi, Girolamo Ranieri, Ettore Cicinelli, Gennaro Cormio

**Affiliations:** 1 Department of Tissues and Organs Transplantation and Cellular Therapies, D.E.O.T., University of Bari “Aldo Moro”, Piazza G. Cesare, 11-Policlinico 70124 Bari, Italy; 2 Department of Biosciences, Biotechnologies and Biopharmaceutics, University of Bari “Aldo Moro”, Via E. Orabona, 4, 70125 Bari, Italy; mengaalessio@gmail.com (A.M.); alessandra.castegna@uniba.it (A.C.); 3 Department of Biomedical and Human Oncological Science, 2nd Unit of Obstetrics and Gynecology, University of Bari “Aldo Moro”, Piazza G. Cesare, 11-Policlinico 70124 Bari, Italy; vera.loizzi@uniba.it (V.L.); ettore.cicinelli@uniba.it (E.C.); gennaro.cormio@uniba.it (G.C.); 4 Interventional Oncology Unit with Integrate Section of Translational Medical Oncology, IRCCS, Istituto Tumori Giovanni Paolo II, 70124 Bari, Italy; 5 Gynaecologic Oncology Unit, IRCCS, Istituto Tumori Giovanni Paolo II, 70142 Bari, Italy

**Keywords:** pericytes, fibroblasts, tumor-associated macrophages, lymphocytes, estrogens, Human Papilloma Virus, ovarian cancer, endometrial cancer, cervical cancer

## Abstract

The tumor microenvironment plays a pillar role in the progression and the distance dissemination of cancer cells in the main malignancies affecting women—epithelial ovarian cancer, endometrial cancer and cervical cancer. Their *milieu* acquires specific properties thanks to intense crosstalk between stromal and cancer cells, leading to a vicious circle. Fibroblasts, pericytes, lymphocytes and tumor associated-macrophages orchestrate most of the biological pathways. In epithelial ovarian cancer, high rates of activated pericytes determine a poorer prognosis, defining a common signature promoting ovarian cancer proliferation, local invasion and distant spread. Mesenchymal cells also release chemokines and cytokines under hormonal influence, such as estrogens that drive most of the endometrial cancers. Interestingly, the architecture of the cervical cancer *milieu* is shaped by the synergy of high-risk Human Papilloma Virus oncoproteins and the activity of stromal estrogen receptor α. Lymphocytes represent a shield against cancer cells but some cell subpopulation could lead to immunosuppression, tumor growth and dissemination. Cytotoxic tumor infiltrating lymphocytes can be eluded by over-adapted cancer cells in a scenario of immune-tolerance driven by T-regulatory cells. Therefore, the tumor microenvironment has a high translational potential offering many targets for biological and immunological therapies.

## 1. Introduction

The tumor microenvironment plays a pillar role in the invasion, progression and subsequent dissemination of cancer cells. Considering the most common gynecological malignancies, the pathogenic process of these neoplasms includes intense crosstalk between the stromal and the cancer cells leading to the acquirement of specific pathological properties. 

In Epithelial Ovarian Cancer (EOC) malignant cells are associated with many other different ancillary cells that are present within the reactive stroma—namely pericytes, adipocytes, endothelial cells, fibroblasts and myofibroblasts, bone-marrow-derived mesenchymal stem cells (BM-MSCs), mesothelial cells and leucocytes [1,2,3]. The genetic expression cluster of reactive stromal signature, described by the Australian Ovarian Cancer Study Group (AOCS), predicts a poorer prognosis and correlates with high levels of desmoplasia and Alpha Smooth Muscle Actin (αSMA) expression by tumor-associated myofibroblasts and pericytes [3,4]. This genetic signature reflects the presence of activated myofibroblasts, vascular endothelial cells, pericytes and the expression of proteins belonging to the extracellular matrix (ECM) reassessment, the cell crosstalk, cell-to-cell adhesion and neo-angiogenesis [4]. On the contrary, an immune signature defines a genetic profile of ovarian cancers with a high rate of infiltrating lymphocytes within tumoral islets that leads to a better outcome [4]. Moreover, the peritoneal cavity that is considered the typical habitat for ovarian cancer lesions, during the development of EOC, is reached by a fluid considered a “dynamic stroma” in which many different soluble factors are present. This fluid *milieu* is empowered by a complex “secretome,” thanks to biological pathways and epigenetic processes [1].

Considering the endometrium, the stromal population around the endometrial glands is characterized by a critical juxtacrine and paracrine activity of estrogen receptor α (ERα), encoded by the gene ESR1, which mediates the release of various growth-factors and cell-cycle-related proteins. This hormonal influence can be enhanced by the dysregulation of other pathways such as E-cadherin loss and mutations of β-catenin, also orchestrating in some cases an epithelial-mesenchymal transition (EMT) during carcinogenesis [5,6,7].

On the other hand, the opposite transition, that is, from mesenchymal to epithelium, has been demonstrated in an animal model of cervical cancer; both Human Papilloma Virus (HPV) and estrogenic significantly influence the stromal cells that are enriched with the paracrine release of pro-inflammatory, mitogenic and antiapoptotic factors [8,9]. Moreover, the fibroblasts surrounding HPV-infected cervical cell can be instructed to produce chemokine, C-C motif, ligand (CCL) 20 to chemoattract T-helper 17 (Th17) lymphocytes [10].

The present review will outline its involvement in the most frequent malignancies affecting women worldwide: epithelial ovarian cancer, endometrial cancer and cervical cancer. This evidence might lead by the near future to the application of target therapies and immunological treatments that will focus on the peculiar biological signature characterizing not only the cancer cells but also their vital microenvironment. 

## 2. Epithelial Ovarian Cancer

EOC is the deadliest cancer of the genital tract, characterized by a significant relapse rate and poor overall survival (OS), mainly because of the usual high stage at diagnosis, which often requires a demanding surgery and the necessity of adjuvant chemotherapies [2,11].

The malignant cells are supplied by a peculiar microenvironment along and through the peritoneal lining, washed by the fluid flow into the abdominal cavity: this physio-pathological feature facilitates the seeding of secondary invasive lesions from the ovarian site without any anatomical barrier [1]. The EOC’s stroma is so crucial for progression and metastatic spread of tumor cells that recent studies have identified many markers for the different tumor-related cells that are able to predict the prognosis [2,3,11]. The various subpopulations of cells and the molecules of the ECM in the EOC milieu contribute significantly to the accomplishment of the cancer dissemination capabilities, as it has been described by Hanahan and Weinberg [12]. 

Therefore, it seems important to consider not only the histological subtype but also the heterogenicity of the cancer microenvironment in the aim of better diagnosis and consequently more efficient therapy. 

### 2.1. Alpha Smooth Muscle Actin (α-SMA) and Platelet Derived Growth Factor Beta Receptor (PDGFβR) *Related Stroma*

#### 2.1.1. Tumor Growth and Metastasis Induction

High levels of desmoplasia and Alpha Smooth Muscle Actin (αSMA) expression, typical of tumor-associated myofibroblasts and pericytes, characterize the reactive stromal genotype of EOC, described by AOCS [3,4]. This genetic signature determines the deposit of a great number of collagen fibers and activated myofibroblasts (desmoplasia) but also the recruitment of vascular endothelial cell and pericytes. Moreover, the activated stroma profile depends on the expression of proteins belonging to the extracellular matrix reassessment, the cell crosstalk, cell-to-cell adhesion and neo-angiogenesis [4]. Among the EOC stroma, Cancer-Associated Fibroblasts (CAFs) are diffusely present and they take origin from different cell lines. Therefore, CAFs can exhibit different markers depending on the derivation from local fibroblast stem cells, bone marrow-derived progenitors or even epithelial components [13]. Consequently, the heterogeneous family of CAFs can be characterized by α-SMA, Platelet Derived Growth Factor Beta Receptor (PDGFβR), podoplanin or FAP (Fibroblast Activation Protein). Various biological signatures could represent peculiar functions and characteristics that enable CAFs to help cancer cells in migrating from primary sites and invading new tissues [2,13]. Notably, some surveys, including the AOCS, revealed the presence of around 150 genetic common markers typical for CAFs or BM-MSCs and normal pericytes, such as PDGFβR, α-SMA, regulator of G-protein signaling 5, caldesmon 1, Melanoma Cell Adhesion Molecule/Cluster of Differentiation 146 (MCAM/CD146), Cluster of Differentiation (CD) 73, angiopoietin 1, Fibroblast Growth Factor (FGF), Fibroblast Growth Factor Receptor (FGFR) and Notch associated molecules [3]. In details, it has been described that normal pericytes usually have decreased levels of α-SMA, so they form a contractile layer enveloping the endothelium of capillaries and venules. The recruitment of pericytes in the tumor growth and subsequent dissemination, depend on both PDGFβR and its endothelial-derived ligand (PDGFβ) and so it can be detected by the new expression of α-SMA. Furthermore, it is known that inhibiting the pathway of PDGFβR in the CAFs population slows the tumor growth [2]. CAFs can be activated by the presence of TGF-β that induces the over-expression of the versican gene (VCAN) upregulating, in turn, the transcription factor NF-κB [14]. The consequent molecular cascade promotes the enhancement of EOC cells’ motility leading to higher chances of overgrowth and invasion [14]. However, a recent in vivo study performed on nude mice in which metastatic serous ovarian cancer (OVAR-5) were injected subcutaneously before chemotherapy, demonstrated the key role of pericytes more than CAFs in bursting the tumor burden and promoting aggressive outgrowth. There were three groups under study: OVAR-5 alone; OVAR-5 with pericytes, OVAR-5 with fibroblasts. The last two groups of animals displayed a tumoral/stromal cells ratio of 10:1 [3]. Strikingly, in the second group, tumoral growth and load increased significantly in a directly proportional way at the rising of pericytes rate. Additionally, also the local invasiveness of the second group arose, leading to an invasive growth beyond the boundaries of the initial tumor mass that was visible to the naked eye [3]. Interestingly, the same study analyzed that the speed and the distance of metastatic seeding of OVCAR-5 cells increased following a direct correlation with the pericytes rate, whereas the controls invaded only the nearest sites to the primary the lesion, for example, bowel and peritoneum. Even though the pericytes rate is directly related to tumor growth and distant dissemination, it has been demonstrated that pericytes can attract other stromal α-SMA^+^ cells without an actual proliferation, tested with the immunohistochemical assessment of the cell-proliferation antigen Ki67 [3,15]. Notably, the pericytes promoted invasion and metastatic dissemination also in a non-metastatic ovarian cancer model (OVCAR-8) [3]. In the end, we could state that activated pericytes and CAFs share a common signature promoting ovarian cancer proliferation, local invasion and distant spread but the former ones behave more powerfully and efficiently.

#### 2.1.2. Pericytes Draw the Architecture of Further Functional Vessels

Both CAFs and pericytes are involved in neo-angiogenesis of EOC, even though the leading role in this process is held by leukocytes. CAFs are responsible for the release of many pro-angiogenic molecules, such as VEGF-A (vascular endothelial growth factor-A) induced by the action of prostaglandin E2 and bradykinin B2. Moreover, the expression of FGF type 2, also known as basic FGF [16], is promoted by the stimulation of CAFs mediated by PDGF or chemokine C-X-C motif ligand (CXCL) [14]. In a breast cancer model, CAFs induce the chemoattraction of the endothelial cells precursors: their structure and function are shaped on the guise of pilot sheets of αSMA^+^ pericytes recruited in the cancer stroma by the PDGFβ signaling [12,17]. In detail, endothelial cells build new blood vessels in the path of the pericytes’ scaffold under the effect of a pro-angiogenic scenario within the TME, orchestrated by a huge variety of classical pro-angiogenic factors such as VEGF, FGF-2, PDGF, angiotensin, Insulin-like Growth Factor (IGF), Tumor Necrosis Factor (TNF), IL-6 and non-classical ones (i.e., tryptase and chymase) [18]. Consequently, both endothelial cells and pericytes undergo the up-regulation of many biological cascades involved in angiogenesis, tumor growth, local invasion and metastasis, as follows MAPK, phospholipase Cγ (PLCγ), Src, Janus kinase (JAK)/Signal Transducer and Activator of Transcription (STAT) and phosphoinositide 3 kinase (PI3K) [18].

Recently, an in vivo study evaluated two models of the angiogenic process in the peritoneal EOC islands using two intraperitoneally growing cell lines grafts in mouse, named IGROV-1 (human high grade serous ovarian carcinoma xenografts) and the ID8 syngeneic mouse ovarian cancer model both labeled by the expression of a lentivirus, eGFP [17]. Researchers observed that after 3 weeks IGROV-1 peritoneal seeds started to show an initial vascularization within the mesenteric spaces reaching definitive and complete viable blood vessels by 6 weeks in all tumor islands sized more the 100 μm. The same experiment was applied to the ID8 model, showing the presence of the first blood vessel between 4 and 10 weeks after the cancer cell intraperitoneal injection. Furthermore, subsequent steps of neo-angiogenesis were studied through confocal microscopy with the help of specific staining for functional blood vessels, CAFs and α-SMA^+^ pericytes knowing that EOC cells grafts were positive for eGFP. They observed that α-SMA^+^ pericytes’ layers were identifiable since the early phase of neo-angiogenesis of avascular peritoneal deposits, but they remained stable as a sort of basements for the future vessels’ walls created by the endothelium [17]. However, Sinha et al. showed that the pericyte coverage index of CD34^+^ blood vessels was the same between controls and graft tissues co-injected with pericytes [3]. Thus, the recruitment of α-SMA ^+^ cells to the Tumor Micro-Environment (TME) of EOC graft model does not affect the possibility for the malignant cells to chemoattract and activate other mesenchymal cells for blood supply. Notably, the eradication of the pericytes population from the TME might determine a wide range of destabilizing effects because of a partial decrease in tumor vasculature as it happens during the treatment with AX102 (an inhibitor of PDGF-β signaling) or in mice holding a mutation in the PDGF-β retention, causing a mild reduction in the number of pericytes [3]. 

#### 2.1.3. Poor Prognosis and Translational Relevance

The first clinical evidence of molecular alterations of TME was given by Corvigno et al., who demonstrated that high PDGFβR^+^ stroma fraction correlates with a poorer outcome even after a statistical adjustment for clinical prognostic features [2]. This marker characterizes CAFs but also pericytes, even though the latter ones are better identified by αSMA when associated with tumors [2]. 

Experimental studies have demonstrated that the PDGFβR pathway is involved in the recruitment and activation of CAFs, leading to tumor growth, dissemination and chemoresistance. Therefore, the inhibition of the PDGRFβ signaling may be critical to overcome some of these features and assess new target therapies against TME elements [2]. However, Sinha et al. demonstrated that pericyte-score, a pericyte-specific gene signature identified by AOCS, correlates with αSMA^+^ cells’ rate. Furthermore, this pericyte-score behaves as a strong predictor for early relapse and increased mortality with reduction of progression-free survival (PFS) and overall survival (OS) [3]. In particular, the mean PFS rockets from 9 to 29 months in case of low pericytes score. Moreover, in the early relapsed group there is higher gene expression, enhanced by pericytes, that promotes cell proliferation, migration, cell motility and reduces cell-to-cell adhesion without affecting significantly angiogenesis in EOC [3]. 

The pillar role of pericytes could be hidden by the presence of many co-expressed markers in common with CAFs and BM-MSCs, such as αSMA, MCAM/CD146 and CD73. Moreover, Sinha et al. showed that even a small number of pericytes co-injected with the tumor graft in mice can influence the TME and consequently the EOC behavior thanks to the recruitment of host αSMA^+^ stromal and Stem Cell Antigen 1^+^/CD73^+^ BM-MSCs [3]. These mesenchymal cells within the TME predict poor prognosis and metastasis, even in the case of OVCAR-8 graft [3].

A future goal for translational research could be the identification of all the signaling molecules released by pericytes to induce tumor growth, dissemination and early relapse. Thence, these proteins may be used as next targets for biological therapies in the EOC *scenario*.

### 2.2. Leucocytes

#### 2.2.1. Imbalance of the Lymphocytes’ Subtypes Correlates with immunosuppressive Environment and Angiogenesis

Lymphocytes represent the shield against cancer cells but some cell subpopulations could hold negative effects on tumors’ pathophysiology, leading to immunosuppression, tumor growth and dissemination. In the EOC *milieu*, we can distinguish a variety of immune cells, which create a complex network in the peritumoral stroma, mainly between the epithelial cells. T cells, identified by the constitutive subunit CD3^+^, represent the main percentage of all CD45^+^ cells within tumoral islets, varying from 35 to 55% in EOC [19]. Once away from the bloodstream, natural killer (NK) cells, T-cells, B-cells, mast cells and macrophages migrate into the peritumoral stroma and EOC islets, within the space between epithelial cells [20].

TILs, an abbreviation that indicates tumor-infiltrating lymphocytes, are involved in the remodeling process because they can mediate immune editing of cancer cells in three main steps: elimination, equilibrium and escape. Firstly, TILs that belong to both innate and adaptive immune system chase after tumoral cells and fight against them to inhibit the building of cancer mass. Next, the remnant EOC cells survive in peculiar homeostasis with their “hunters” because of two main reasons: a variety of stronger phenotypes acquired by tumoral cells and a different microenvironment of adaptive immune cells, balanced mainly by cytotoxic CD8^+^ lymphocytes and CD4^+^CD25^+^ T-regulatory cells (T_regs_), responsible for antigen self-tolerance. Finally, cancer cells become skilled at immune *camouflage*, realizing one of the most powerful tumor hallmarks that consist of escaping from antigen recognition and killing [12,13]. At this point, the tumoral mass becomes clinically relevant [21,22]. It can be concluded that inflammatory cells, mostly the cytotoxic ones, play a pivotal role in the tumor growth restriction but they can be eluded and partly defeated by over-adapted cancer cells and the consequent stroma remodeling. 

The benefits of TILs presence within EOC is mainly pronounced for CD8^+^ lymphocytes especially when they co-express the integrin α_E_ (CD103)/ β_7_. This is a typical marker of intraepithelial lymphocytes of regular skin and intestinal mucosa, even though the bloodstream lacks of CD103^+^ T cells (since they account just for 2%). Notably, CD103 acts only as a receptor for E-cadherin covering epithelial cells’ outer membrane. Consequently, its axis anchors T cells to healthy and tumoral epithelium causing a forced homing of immune cells, specific for local tissue antigens.

EOCs stands for a variety of different malignancies with different biology and behavior even deeply in their microenvironment: the highest mean rate of CD103^+^ has been found in serous histological subtype, then endometrioid followed by clear cell and mucinous [23]. However, the positive prognostic value of CD8^+^ CD103^+^, evaluated as the percentage of Specific Disease survival, was statistically significant only in High Serous Grade Carcinoma (HSGC), followed by mucinous [23]. Despite this, the pivotal role of CD103^+^CD8^+^ T cells and CD103^+^ NK cells in juggling the intraepithelial immune response and managing tumor overgrowth emerged strongly in HGSC. In other words, CD103 acts as a marker for “tissue-resident memory” on the surface of CD8^+^ T cells (CD8^+^ TRM) and determines a good prognosis for patients with EOC [23]. Among cytotoxic lymphocytes, CD8^+^ T cells need the action of helper cells and the Human Leucocyte Antigen (HLA) system, whereas NK lymphocytes become active after the direct contact with cancer cells. In the last years, different studies produced numerous evidence about different lymphocytes’ subpopulations. Firstly, it has been demonstrated that Class I HLA expression correlates with the density of CD8^+^ TILs and induces their activation and proliferation in the presence of interleukin (IL) 15 and 17 [20]. Moreover, islets of tumor rich of CD3^+^/CD8^+^ TILs show a high mRNA level of interferon γ (INF γ) and IL-2, secreted by CD4^+^ T-helper 1 cells and activated CD8^+^ T cells [19,24]. Furthermore, TEM with TILs is also positive for macrophage-derived chemokines and secondary lymphoid-tissue chemokine proteins. Therefore, there is a strong positive correlation between the number of CD4^+^ and CD8^+^ among TILs in EOC (*p*-value < 0.001). On the contrary, these chemokines were undetectable as mRNA in case of EOCs without T-cells infiltration [19,24]. 

In details, the function of INFγ is critical in the EOC microenvironment, even in the ascites and peritoneal dissemination seeds, showing a positive correlation with the CD8^+^ TILs’ concentration. However, higher levels of INFγ induce the expression of PD- Ligand 1 (PD-L1) on ovarian cancer cells and inhibit CD8^+^ T cells via PD-1, the relative matching receptor. Once the effector T cells are blocked by PD-1/PD-L1 axis, they cannot sustain the cell cycle steps and consequently, they undergo apoptosis [19,24]. A similar negative function belongs to Cytotoxic T-Lymphocyte-Associated Protein 4 (CTL4) because of the check-point blockade in effector T cells [20]. 

Activated cytotoxic CD8^+^ lymphocytes share their tumoral islet spaces also with oligoclonal CD20^+^ B lymphocytes, which have already experienced antigen recognition and subsequent class-switching for Ig-G [25]. Interestingly, their antibodies differ from the serum ones that recognize usual cancer antigens, such as TP53 or NY-ESO-1. Since CD20^+^ TILs have no impact on the humoral response, they might join the cellular immunity helping the CD8^+^ subset. Nielsen et al. (2012) found that CD20^+^ cells, which occupy intraepithelial spaces and peritumoral stroma, colocalize with CD8^+^ in EOC, improving survival at a higher rate than CD8^+^ alone. Moreover, CD20^+^ TILs are positive for typical Antigen-Presenting-Cells (APCs), such as MHC Class I and II, CD80, CD86, and CD40. An intriguing hypothesis explaining this finding is that B cells may prevent cytotoxic cells’ anergy, playing the role of a stimulation *reservoir* for a persistent tumor lysis activity [25].

The immune response against EOC is balanced by the inhibiting function of T_regs_, which is characterized by the expression of the forkhead box P3 (FOXP3) and two peculiar clusters of differentiation, namely CD4 and CD25. It is well known that T_regs_ alter the EOC *milieu* restraining the ability of the immune system to destroy cancer cells through the release of inhibitory cytokines, mostly Tumor Grow Factor β (TGF-β) and IL-10, and/or thanks to a direct cell-to-cell block [20]. A population of regulatory cells is fundamental under a regular situation but in an oncological setting it reduces the chances to survive because of an undesirable “self-tolerance.” The activation trigger for T_regs_ is represented by the presence of CCL28 that arises under hypoxia condition, in such a way that the tumor overgrowth settles a vicious cycle [20]. The population of T_regs_ increases in case of TILs enriched with B7H4^+^ tumor-associated macrophages (TAMs) contributing to reducing the outcome. Other negative actors in the immune microenvironment of EOC are represented by up-regulated vascular endothelial growth factor (VEGF), a pro-angiogenic but also an immunosuppressive factor, and endothelin-B that reduces the permeability of tumoral blood vessels, the lymphocytes diapedesis and, as a consequence, their possibility to contrast the cancer cells [20]. TME can be turned into a pro-angiogenic set by the interplay between cancer cells and stromal ones, mainly leucocytes. The main sources of pro-angiogenic factors, classical (i.e., VEGF) or non-classical ones (i.e., tryptase), are macrophages and mast cells under hypoxic conditions [18,26]. 

#### 2.2.2. Imbalance of the Lymphocytes’ Subtypes Correlates with Poor Prognosis but Harbors a Highly Critical Translational Significance

The presence of TILs represents a critical signature for the prognosis of patients with EOC, as demonstrated by many recent studies. The CD3^+^ inflammatory *milieu* is present in 55 % of EOCs and it is responsible for a significantly higher 5-year survival (38.0%) compared to the absence of TILs (4.5%). Notably, the median PFS in case of T-cells populations within tumoral islets is 22.4 months and the median OS accounts for 50.3 months; on the other hand, their values plummet to 5.8 and 18 months, respectively in EOCs without T-lymphocytes (*p*-value < 0.001 for both comparisons) [19]. Similar results involve the subgroup of patients with complete response after optimal debulking surgery and adjuvant chemotherapy based on platinum: people with the evidence of T cells’ presence at the pathological exam have a 5-year OS rate of 73.9%, whereas the others without T-cell TILs have just an 11.9% OS rate [19]. However, a multivariate analysis showed that optimal debulking surgery and the presence of TILs behave as independent good prognostic factors for EOC [19]. 

The main role is played by CD8^+^ T cells, especially when they carry also CD103 as memory antigen and in case of colocalization with CD20^+^ B cells, leading to a survival rocketing. Even though CD8^+^ TILs can infiltrate every EOC’s histological type, their prognostic benefit is more evident in HGSC [19,23,25]. Since VEGF reduces the number of TILs, it is associated with a poorer outcome as in case of the low rate of INFγ, IL-2, and lymphocyte-attracting chemokines. In details, the up-regulation of VEGF correlates with early recurrence (within 6 months; odds ratio = 0.34; area under the curve = 0.80; *p*-value = 0.05). On the contrary, overexpression of macrophage-derived chemokine predicts late recur [19]. Similarly, endothelin prevents T-cells from homing within tumoral islets and consequently it mines the survival possibilities. Therefore, it has been demonstrated that knock-down mouse for endothelin presented an increased population of TILs [20]. 

CD8^+^ lymphocytes and CD4^+^ T_regs_ are considered as two contrasting subpopulations of T-cells among TILs: the former ones predict a longer survival, whereas the latter ones correlate with immune suppression and poor survival in solid tumors. The ratio CD4^+^FOXP3C^+^ T_regs_/CD8^+^ T cells rises when the tumor contains also B7H4^+^ TAMs, leading to decreased cancer lysis and poorer outcome. Thus, the possibility of obtaining a T_regs_ depletion and/or more easily converting them into T helper 17, in the presence of IL2, might be a therapeutic perspective [20]. Aoki et al. (1991) used IL2 in the first human adoptive TILs for the therapy of advanced or recurrent EOC: autologous CD8^+^ TILs taken form pleural effusion were cultured with IL2, expanded and then infused with or without chemotherapy [27]. As a result, 7 patients in 10 who received both TILs and chemotherapy had a complete response, whereas in the other group the complete response accounted just for one case among seven patients [27]. Unfortunately, this kind of immunotherapy has many pitfalls and it can fail because of the typical HLA downregulation acquired by EOC to evade the T cells recognition. Consequently, their infiltration within the tumor is reduced as the adoptive TILs therapy efficacy [20]. An interesting preclinical study on mouse EOC model showed that the contemporary inhibition of CTLA-4 and PD-1, associated with vaccination, determined the rejection of the tumor [28]. Furthermore, phase II clinical studies demonstrated encouraging results for anti-PD-1 and CTLA-4 but more studies are needed to confirm the data. Since CD137^+^ TILs have strong activity against tumor overgrowth, some preclinical studies introduced the use of the agonistic antibodies specific for CD137 with a positive outcome [20]. Effective immunotherapy is also the inhibition of the negative immune-modulator, precisely T-cell immunoglobulin and mucin-domain-containing-3 (TIM3), that is significantly enhanced by the combination with agonistic CD137 antibodies in murine models leading to 60% of cases free of tumor in 90 days from cancer inoculation [29]. An intriguing possibility could be the use of genetically modified T cells with a chimeric T cell receptor restricted for HLA-A2 that can bind a specific epitope of HER2, a typical EOC antigen [20]. 

Moreover, almost all EOC but not healthy epithelium, express Major histocompatibility complex class I-related chains (MIC) A and B and UL-16 binding proteins (ULBPs), ligands of Natural killer group 2, member D (NKG2D) that characterizes NK cells, CD8^+^ cytotoxic T cells and γδ-T cells [30]. Even though the stimulation of NKG2D activates the cytotoxic activities of T cells, a high expression of ULBP2 reduces the TILs number and correlates significantly with lower survival, rather than MIC A/B [30]. Notably, ULBP2 can be processed by matrix enzymes and released in the bloodstream inducing a probable central down-regulation of NKG2D expression that prevents CD8^+^ and CD57^+^ cells from homing to tumoral islets and destroy them, as in a homeostatic system [30]. A hypothetical solution to restrain the process of TILs disruption may be a neutral competitor ligand for NKG2D or anti-ULBP2 to create an artificial balance without impairing the cytotoxic activity of T cells within the tumor.

#### 2.2.3. The Role of TAMs in EOC

Macrophages are phagocytic cells of the innate *immune system* present in almost all tissues. They are a very diverse set of cells, able to significantly modify their activity in response to external stimuli. They undergo a polarization process with the expression of surface markers and the acquisition of functional states depending on the stimulating factors such as cytokines and other signals. According to the traditional concept of binary polarization, the phenotypes of activated macrophages are conventionally divided in the M1 and M2 category [31,32]. Classically activated macrophages—also known as M1 macrophages—are polarized by inflammatory signals such as IFNγ and lipopolysaccharides (LPS), and exhibit bactericidal, immunostimulatory and antitumoral activities. By contrast, alternatively activated macrophages or M2 macrophages are polarized by anti-inflammatory signals, such as IL10, IL4, and IL13 and are involved in immunosuppression, tumor invasion, tumor growth, angiogenesis and metastasis [33,34,35]. Tumorigenesis is a complex phenomenon in which macrophages, also known as TAMs, play an important role as main components of the TME. During cancer progression TAMs reach a polarization state resembling that of M2 macrophages, as several M2 markers (such as CD163, CD206, PD-L1, ARG1) progressively increase [34,36], although it is becoming evident that TAMs exist in a variety of polarization states which are far from being fully elucidated [37]. 

In the context of the TME, many stimuli are provided by cancer cells. The EOC cells, for instance, are able to polarize macrophages toward a tumor-associated phenotype [38] by secreting several factors, such as the colony-stimulating factor 1 (CSF-1), which acts as both a chemoattractant and a mitogen for circulating monocytes via tyrosine kinase receptor CSF1-R binding [39]. It is now evident that ovarian TAMs, in response to a plethora of soluble mediators of the TME, adopt a mixed alternatively and classical phenotype, which is functionally associated to defective antitumoral functions including impaired antibody-dependent cell-mediated cytotoxicity and phagocytosis [40,41]. Genome-wide expression analysis of TAMs isolated from HGSC shows that few M2 markers such as CD163 and IL-10 are upregulated, together with the M1 markers CD86 and TNFα, compared to non-polarized (M0) macrophages [41,42]. Evaluation of M1 (HLA-DR, iNOS) and M2-polarization (CD163, VEGF) markers in TAMs from EOC patients concluded that an increased M1/M2 ratio is associated with better overall survival [43,44]. In line with these findings, a higher ratio of CD163^+^ of CD68^+^ (CD163/CD68) TAMs and higher serum levels of CD163 correlate with higher tumor grade, worse PFS and early relapse of serous ovarian carcinoma after first-line therapy [45,46,47,48,49]. In addition, the expression of the alternative activation marker CD163 in TAMs strongly correlates with elevated IL6, IL10, TGFβ, and polyunsaturated fatty acids (PUFAs) levels, in particular, arachidonic acid, which are signaling mediators and/or prognostic markers in ovarian cancer at different levels [41,50,51,52,53,54,55]. Similarly to CD163, the M2 marker CD206 is associated with poor prognosis in TAMs from ovarian cancer [56]. 

**Figure 1:**
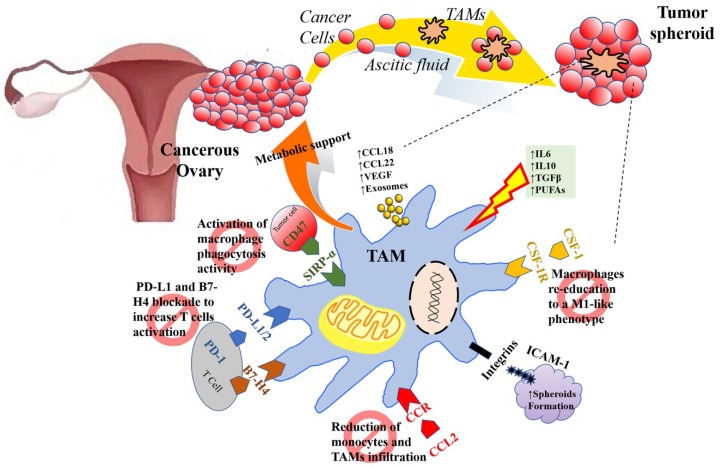
Tumor-associated macrophage (TAM) function in the ovarian cancer tumor microenvironment (TME) and examples of therapeutic strategies. Multiple mediators (such as IL10, IL6, TGFβ, PUFAs, etc.) in the TME determine the activation state and function of TAMs. In response to these triggers, TAMs produce a plethora of tumor-promoting soluble factors (such as VEGF, CCL22, CCL18, etc.) and extracellular vehicles (Evs) and probably supply metabolic support to cancer cells. TAMs provide matrix support and growth factors also in the core of the tumor spheroids, which are stabilized by ICAM-1. During ovarian cancer progression, tumor cells detach from the primary tumor and interact with TAMs to survive in the ascitic fluid as free-floating spheroids. Blocking key macrophage pathways influences the tumorigenic and immunosuppressive activities of TAMs, providing tools for the development of novel therapies to be combined with classical chemotherapy for gynecologic cancers. Inhibiting the PD-L1/2 immune checkpoint pathway results in reactivation of T cells. Re-activation of phagocytosis and promotion of M2 to M1-like phenotype shift are achieved by inhibiting the CD47 and CSF-1R pathways, respectively. Furthermore, inhibitors of chemokines (such as CCL2) involved in the recruitment of monocytes can prevent TAMs differentiation and accumulation within the TME, thereby reducing tumor growth and dissemination.

An overall view of the main TAMs functions in the EOC TME and their translational perspectives areillustrated in Figure 1.

From a functional point of view, TAMs play important functions in sustaining EOC diffusion. This is partly related to the fact that the EOC TME extents into the peritoneal fluid, which at advanced stages occur as a malignant effusion (ascites). This fluid facilitates cancer cell dissemination throughout the peritoneal cavity since it contains detached tumor cells, tumor cell spheroids, different types of innate and adaptive immune cells, as well as a plethora of tumor-promoting soluble factors and extracellular vehicles (EVs) [57]. Several studies have demonstrated that ovarian TAMs increase in number during cancer spread [39,58,59] to the point that 50% of cells in the peritoneal TME and malignant ascites can be represented by TAMs [45], which release protumorigenic and immunosuppressive factors. 

TAMs sustain metastasis dissemination through CCL18 secretion [60] and enable the trafficking of immune-suppressive T_regs_ to the ovarian tumors through CCL22 secretion [61]. Furthermore, TAMs contribute to angiogenesis by VEGF production [62] and suppress T-cell cytotoxicity by expressing both the coinhibitory molecule B7-H4 [63,64] and the PD-1 ligand PD-L1 at a higher level than EOC cells, resulting in T cell exhaustion and inactivation of cytotoxic T cell responses [65]. It has been recently demonstrated that miRNAs (miR-29a-3p and miR-21-5p) transferred from TAMs to CD4^+^ T cells through exosomes synergistically induce the immunosuppressive (T_regs_)/proinflammatory (Th17) T-cell imbalance, promoting an immune-suppressive microenvironment [66].

Another way by which TAMs support cancer cell dissemination is through favoring spheroid formation [67]. During EOC progression, tumor cells detach from the primary tumor and interact with TAMs to survive in the peritoneal fluid as free-floating spheroids. TAMs provide matrix support and growth factors in the core of the tumor spheroid in the initial steps of peritoneal carcinomatosis. TAMs secrete large amounts of EGF to activate the EGF receptor (EGFR) in surrounding tumor cells, thereby supporting tumor proliferation and anoikis protection [62]. The EGF/EGFR TAM-tumor cell crosstalk induces autocrine VEGF-C/VEGFR-3 signaling in tumor cells, which upregulates integrins and ICAM-1 necessary for maintaining cell-to-cell contact between tumor cells and TAMs (via CD11b/c) and stabilizing the tumor spheroids with (chemo)protective properties, as suggested by Freire et al. [62]. The close interaction between tumor cells and TAMs might go beyond a “physical” interaction and be characterized by a metabolic cross-talk. For example, high-invasive, aggressive and resistant ovarian cancer cells are addicted to glutamine, with high glutaminase activity and low glutamine synthesizing capacity compared to low-invasive cells [68]. The glutamine addiction of cancer cells might induce extracellular glutamine depletion, which is known to increase glutamine synthetase expression in macrophages. This metabolic feature in macrophages promotes the acquisition of an immune-suppressive, proangiogenic and premetastatic M2-like polarization state and favors macrophage-produced glutamine secretion [69,70]. By depleting extracellular glutamine, cancer cells might achieve a dual goal, which is the functional and metabolic TAM exploitation [71].

**Figure 2:**
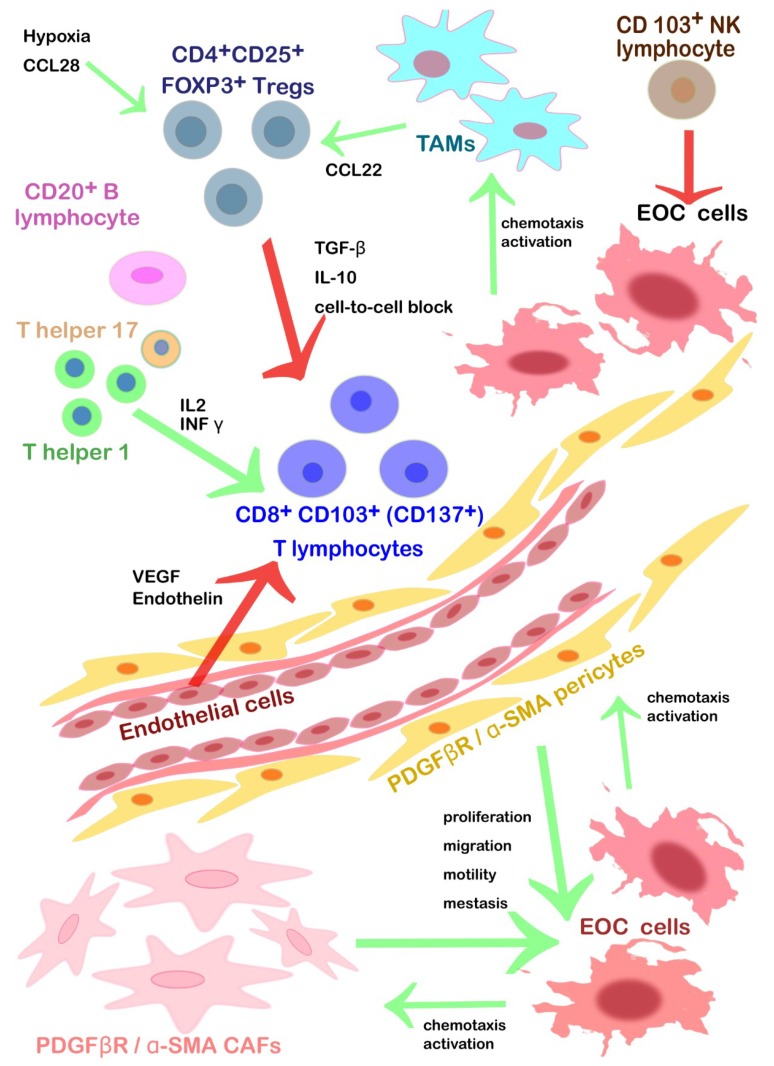
The main cell types that populate the EOC’s TME: overall functions and cross-talks. Within EOC’s tumoral islets, the stroma holds a variety of cell types, such as CAFs and α-SMA+ pericytes. CAFs are recruited by PDGFβ and they promote EOC cells’ motility, overgrowth, neo-angiogenesis, and invasion. α-SMA+ pericytes are recruited by the PDGFβ and they reduce EOC cell-to-cell adhesion without affecting angiogenesis significantly unless providing guide-sheets for endothelial cells during neo-angiogenesis. Their rate and genetic signature correlate with proliferation, migration and cell motility of EOC. CAFs, α-SMA+ pericytes and EOC cells communicate via a mutual cross-talk that creates a vicious cycle towards cancer progression. TILs include CD103^+^ NK lymphocytes, T helper 1 lymphocyte, T helper 17 lymphocytes, CD8^+^ CD103^+^ (CD137^+^) lymphocytes. Monocytes from the bloodstream are chemoattracted by cancer cells and become resident TAMs, activated by IL10, IL6, TGFβ, PUFAs and they play a key role in the EOC’s biology (see Figure 1). Among TILs, there are CD103^+^ NK lymphocytes that kill EOC cells expressing NKG2DLs. CD8^+^ CD103^+^ (also CD137^+^ in most cases) cytotoxic T lymphocytes need to be activated by T helper lymphocytes (type 1 or 17) under the trigger of INF γ and IL-2 to chase after cancer cells and to attack them as effector T cells. Resident CD20^+^ B cells cohabitate with effector T cells and they probably help them as APCs preventing their anergy. The diapedesis of this TILs is inhibited by endothelin and VEGF mainly from the endothelium. Among TILs, there are also CD4^+^CD25^+^ FOXP3^+^T_regs_ that are activated by CCL28 under hypoxia condition and in the presence of B7H4^+^ TAMs. They inhibit the cytotoxic functions of T cells effectors releasing inhibitory cytokines (TGF-β and IL-10) or via a direct cell-to-cell block. The green arrows stand for positive *stimuli*, whereas the red ones represent an inhibition towards the pointed target.

A summary of the main features characterizing the stromal cells within EOC’s TEM and their translational relevance are described in Table 1 and in Figure 2 below. 

## 3. Endometrial Cancer

Endometrial cancer (EC) covers the main percentage of gynecological malignancies in Western countries. Its worldwide incidence accounts for 142000 cases per year with a life-long risk for the diagnosis of 1.71% [72,73]. This cancer affects mainly post-menopause women since the median age at the diagnosis is 63 with an incidence peak in the next decade. Thanks to the early and frequent clinical presentation with abnormal vaginal bleeding, the diagnosis comes usually at initial stages with a good prognosis. Even considering patients with any stage of the disease, they will have a 5-year OS of about 80% [72,73].

EC mostly belongs to type I (80–90%), which is estrogen-dependent and characterized by endometroid histology, early presentation at diagnosis, excellent prognosis, molecular signature, involving mutations of Phosphatase and Tensin homolog (PTEN), KRAS, Catenin Beta 1 (CTNNB1) and phosphoinositide 3 kinase (PI3K), the mismatch repair protein MutL Homolog 1 (MLH1) promoter hypermethylation and autotaxin hyperexpression [73,74]. Type II refers to a rare histological diagnosis (10–20%), namely: serous, clear cell, undifferentiated carcinomas, carcinosarcoma/malignant mixed Müllerian tumor. They are known to recur even at early stages with a lower OS. Even though TP53 mutation is typical of serous EC, it could be identified in ¼ of endometrioid cancers, introducing some criticisms in the classical EC dichotomy. Therefore, the Cancer Genome Atlas (TCGA) Research Network describes four molecular types among endometrial tumors: 1. DNA POLymerase Epsilon (POLE) hypermutated tumors; 2. tumors with microsatellite instability; 3. tumors with a high rate of copy-number mutations, mainly involving TP53; 4. tumors without any of the features listed above. There is also a hereditary EC, typically observed in association with hereditary non-polyposis colon cancer (HNPCC, Lynch syndrome) [73].

The pivotal treatment for EC is total hysterectomy with salpingo-oophorectomy, lymph node dissection or sentinel lymph node biopsy in selected cases, eventually followed by adjuvant radiotherapy according to the risk assessment. Only in advanced stages and special histotypes, such as serous ones, the adjuvant chemotherapy is considered [72,73]. 

Few studies have been conducted about the endometrial stroma focusing mostly on endometrioid ECs. Leukocytes, especially TAMs, as well as fibroblasts and myofibroblasts, compose the cell population under study that plays a crucial role in the malignant progression of hyperplasia leading to the hormonal sensitive EC [5,7,75,76,77,78].

### Mesenchymal Cells and their Autocrine and Paracrine Role in the Development of EC

A recent study isolated and cultivated human endometrial stromal cells after removing all the leukocytes with an anti-CD45: the pillar population is represented by fibroblasts, which are typically positive for CD90, collagen 1 and 5B5. In the presence of PDGF, they can differentiate into myofibroblasts acquiring αSMA positivity. The remaining stroma hosts vascular smooth muscle cells, endothelial cells (CD31) and leukocytes [77].

Novel sensitive markers for endometrial stroma are CD10 or common acute lymphoblastic leukemia antigen and CD225 also named interferon-inducible transmembrane protein 1. They could be useful during the pathological staging assessment, especially in case of troubling diagnosis of myometrium invasion. CD225 is more sensitive (71.4%) and specific (100%) than CD10 in detecting the stromal walls at the endometrial-myometrial junction in endometroid EC [78]. Notably, CD225 is more than a simple marker, because it plays a key role in mediating the antiproliferative and growth-inhibitory effects of INFγ. Since endometrial glands and myometrium lack of CD225, its absence at the correspondent staining indicates that the tumoral cells took the place of stromal ones and passed through the junction between endometrium and myometrium [78]. 

The crucial role of the stroma was recently observed through the effects of the mesenchymal deletion of ERα in a mouse model after bilateral oophorectomy. The treatment with exogenous 17β-estradiol (E2) leads to lower values of Ki67 among epithelial cells when the stroma is ERα deficient. It is known that the E2 pathway activates the ERα of mesenchymal cells leading to the release of growth factors, IGF1 or Tumor Growth Factor (TGF) and cell-cycle-related proteins, such as mitotic arrest deficient 2 like 1 (MAD2L1), Cyclin-Dependent Kinase Inhibitor 1A (CDKN1A) and CCAAT enhancer binding protein beta (CEBPβ) [7]. Thence, in the ESR1 deletion study, the E2-induced cascade of IGF1, MAD2L1, CDKN1A, and CEBPβ was attenuated [7]. In other words, stromal ERα mediates the E2 proliferative stimuli to the adjacent endometrium promoting the paracrine secretion of mesenchymal cells, as mentioned above [7]. Furthermore, IGF1 is the leading growth factor in the malignant progression from Endometrial Hyperplasia EH (EH) to EC in most of the cases, since the 80-90 % of EC is estrogen-dependent [7,72]. The juxtacrine action of stromal cells can also influence the Progesterone Receptor (PR) expression of the neighboring epithelial cells: in case of ESR1 deletion, even during E2 therapy after ovariectomy in mouse, the endometrium had a PR loss [7]. In conclusion, the researchers evidenced the crucial relevance of the stromal ERα in mediating the full potential of the estrogenic proliferative trigger on the endometrial lining and determining its proper PR expression [7].

An interesting study evaluated the paracrine interaction between the peri-glandular stromal cells, identified with the expression CD10 and the epithelium in ECs, EH and controls [6]. The researchers started from the assumption that the mutation of CTNNB1, notoriously associated with endometrioid EC and EH, results in abnormal expression of β-catenin. Its pathway is influenced by the hormonal *milieu* of endometrium since the β-catenin/E-cadherin rate is higher in the post-menopausal age than in pre-menopausal one. Moreover, it increases progressively through the multi-step progression from HE to EC [6]. Conversely, the expression of ER and PR within the stroma tend to decrease in the carcinogenesis process partly due to the influence of the disruptor β-catenin/E-cadherin. Notably, β-catenin and E-cadherin determine the regular architecture of endometrium, assuring the necessary cell-to-cell adhesion to the epithelium. The contemporary loss of E-cadherin and the up-regulation of β-catenin promote the epithelial-mesenchymal transition (EMT) in many types of cancers, including EC [6]. Stromal cells orchestrate the differentiation and the proliferation of the near epithelium components of the uterus, also managing the interaction between the β-catenin and the hormonal paracrine stimuli. Interestingly, the expression of many markers such as β-catenin and EMT-associated proteins (TWIST, SNAIL-SLUG) has the opposite behavior in epithelium and stroma. The peri-glandular stroma expression of β-catenin, SNAIL-SLUG, TWIST, ER and PR decreased significantly in EC compared to HE and in HE compared to controls [6]. Therefore, imbalanced homeostasis within the mesenchymal microenvironment leads the epithelium to lose the cell-to-cell adhesion, with the subsequent acquirement of the ability to escape from apoptosis, migrate and invade in response to ER activation [6]. Consequently, β-catenin and EMT-associated proteins expression levels were significantly higher in EC epithelium than EH and similarly in EH than controls. Conversely, E-cadherin decreased in EC more than EH and in EH compared to controls. This data could depict an interesting *scenario* where sex hormones, β-catenin, and EMT-associated proteins exert a cross-talk with the endometrium in the peritumoral mesenchyme, which contributes to the step-by-step development of EC from EH with atypia, which in turn comes from EH without atypia [6]. 

**Figure 3:**
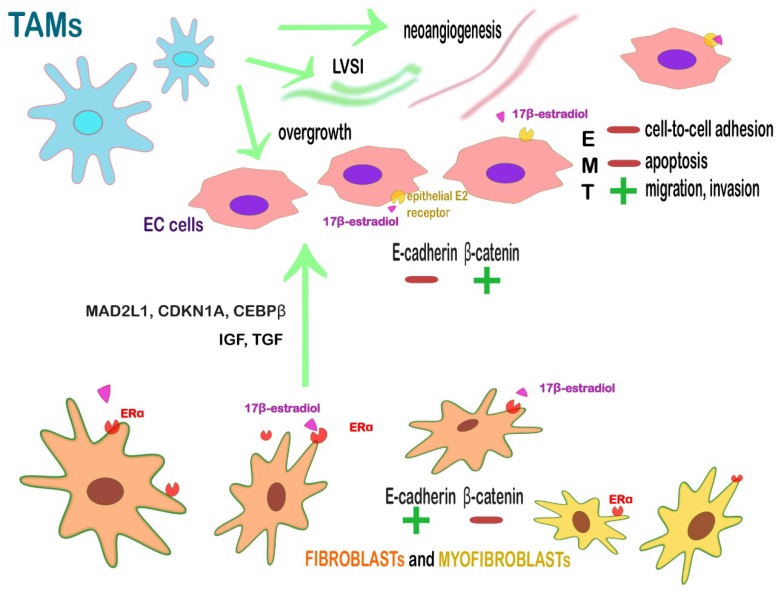
Crosstalk between endometrial cancer’s (EC) cells and the surrounding stroma. The peritumoral stromal cells in EC are mainly composed by ERα+ fibroblasts and myofibroblasts, TAMs and TILs. Fibroblasts and myofibroblasts, activated by the binding 17-β-estradiol and its stromal receptor ERα, secret cell-cycle-related proteins (MAD2L1, CDKN1A, CEBPβ) and growth factors (IGF, TGF). This juxtacrine and paracrine effect leads the epithelium to EMT losing the cell-to-cell adhesion and triggering the ability to escape from apoptosis, migrate and invade. The contemporary loss of E-cadherin and the up-regulation of β-catenin drive the EMT process leading to the alteration of the endometrial architecture and the subsequent multistep process towards EC. Fibroblasts and myofibroblasts act as sentinel and amplifier of estrogens on the neighboring endometrium. They are characterized by an opposite expression of E-cadherin/β-catenin compared to the epithelium (represented as a green positive sign or as red negative one to indicate respectively increase and decrease) and they express αSMA marker. Other important actors within TEM are CD163^+^ M2 TAMs because they promote angiogenesis, LVSI, lymph node metastasis, and tumor overgrowth. The green arrows stand for positive *stimuli*.

An overall view of the stromal cells that surround ECs is illustrated in Table 2 and in Figure 3

## 4. Cervical Cancer

Cervical cancer (CCx) represents the second most common malignancy among women all over the world and causes about 273200 deaths per year [79]. Its incidence is higher in Developing Countries and its roots lie in a gradual cancerous process from precancerous lesions, caused by unremitted high-risk HPV infections, sustained mainly by virus types 16 and 18 [79]. The multistep carcinomatous process could depend on the inefficient immune response within the cervical *milieu*, leading to the persistence of HPV-infected epithelial cells. Therefore, HPV infection, intraepithelial lesions, and their recurrence are more frequent in people with immunodeficiency and in women with a coexistent *Chlamydia trachomatis* infection [80]. HPV is characterized by two powerful oncoproteins, namely E6 and E7, able to downregulate the expression of TP53 and retinoblastoma protein (pRB) respectively leading to the cancer initiation and promotion [9,79]. Recent researches showed the critical role of tumor-associated NK cells as the first shield against microbes along the mucosal lining since they act as cytolytic lymphocytes and they also provide chemo-attractive molecules for other effector leukocytes. Consequently, this innate immune response correlates with a better prognosis in contrast with the EOCs [30,79]. Cancer-promoting effect of the high-risk HPVs works together with a high estrogen level within the local hormonal environment. Both experimental studies on mice and epidemiologic evaluations on a cohort of patients evidenced the crucial role of E2 and its stromal receptor ERα encoded by the gene ESR1 [8,9]. Notwithstanding the pillar role of CCx screening with Papanicolaou test and/or the HPV genotypisationsince their introduction in plummeting incidence and mortality, many women still escape from them. Since the leading cause of CCx is high-risk HPVs, a vaccine against them has now started a virtuous circle in reducing the percentage of HPV-affected young people and consequently in herd-immunity creation. Thence, the future challenge may be the HPV eradication thanks to efficient campaigns in favor of broad-spectrum vaccines [81].

### 4.1. Inflammation, Pro-angiogenesis and Cancer-instructed Stromal Fibroblasts in CCx: Pathways and Translational Relevance

CCx *milieu* can be considered as the final product of the effects of the persistent high-risk infection of HPV on epithelial cells that in turn instruct the surrounding stromal cells within the intercellular matrix. In precancerous and in early stages of cancer HPV oncoproteins modulate the immune response via the inhibition of pro-inflammatory molecular cascade in keratinocytes, mainly reducing the expression of CCL2 also referred as monocyte chemoattractant protein 1 (MCP1) and CCL20, also known as macrophage inflammatory protein-3α (MIP3A) [10]. In the subsequent steps of cancer progression as in High-grade Squamous Intraepithelial Lesion (HSIL), cancer cells mold their stromal neighbors in chemo-attractors for dendritic cells and macrophages via the activation of CCL2/C-C motif Chemokine Receptor (CCR) [10]. 

Notably, higher stages of CCx are associated with an increased expression of two innate immunity ligands, already named before in the sections related to EOC. These are MICA/B and ULBP1 and they rise at the same pace of cancer progression from Low-grade Squamous Intraepithelial Lesion (LSIL) to HSIL and invasive lesions [79]. Both these molecules bind NKG2D, so they are usually referred to as NKG2DLs (ligands). Even though NKG2D is typically found in NK cells, it characterizes also CD8^+^ cytotoxic T cells and γδ-T cells. Screening, treatment, and follow-up of patients with this tumor may benefit from the rising value of NKG2DLs along the multi-step CCx evolution [79]. In other words, they can help in detecting pre-invasive lesions at high risk for cancer progression but they can also predict the kind of immune cells recruitment within the tumor-associated stroma. Since high levels of NKG2DLs reflect increased immune system activity against tumoral cells, recent research showed a strong positive correlation of PFS and OS with MICA and/or ULBP1 [79]. Notably, these findings do not match with the EOC’s ones that identified MICA/B and ULBP2 as poor prognostic factors in EOC [30]. This controversial NKG2DLs’ prognostic effect can be explained by specific cross-talks among different TEMs, such as cervical and ovarian ones, justifying opposite cellular reactions to similar starter actions. In details, CCx *milieu* is influenced by the paracrine activity of tumoral cells and its effect on all the stromal cellular populations, mainly represented by fibroblasts (vimentin^+^) and myofibroblasts (αSMA^+^). The cancer cells enable stromal ones to up-regulate CCL20 thanks to the paracrine trigger of the molecular cascade by the activation of the transcription factor CCAAT enhancer binding protein beta (C/EBPβ) and to the release of IL6 [10]. The latter has a wide range of functions: firstly, it acts as a chronic pro-inflammatory/pro-tumoral agent via the chemoattraction of Th17; secondly, it can be considered a disruptor of APCs helping cancer cells in immunity escape and a cancer promoter thanks to the induction of matrix metalloproteinase 9 (MMP-9) [10].

Therefore, higher rates of CCL20 call for its receptor CCR6 located on the CD4^+^/IL17^+^/CCR6^+^ T lymphocytes, leading to the presence of Th17 TILs [10]. Their number is associated with invasive lesions and increases together with the progression of FIGO stage. Since the deletion of C/EBPβ and the inhibition of IL-6 impair significantly the migration properties of Th17 cells at more than 80% of their original activity, new therapies against this pro-tumorigenic subset may improve the therapies’ options in case of advanced CCx [10]. Thence, paracrine agents like IL6, chemo-attractants like CCL20 or its activation pathway in the CCx-instructed fibroblasts may represent the next targets for personalized therapies in advanced CCx. Moreover, immunotherapy against Th17 can be translated also for CCx, as already done for EOC in the gynecological field. 

The stromal *milieu* of CCx is rich of many other cytokines secreted after local epithelial-mesenchymal cross-talk. Interestingly, recent research discovered that the coculture of human CCx cells and autologous fibroblasts is associated to higher levels of CXCLs, mainly CXCL5 and CXCL1 and their receptor CXCR2 involved in a pro-angiogenic pathway [8]. Fibroblasts also face a massive ECM reorganization driven by HPV oncoproteins, E6 ed E7, that downregulate stroma expression of the fibronectin promoter, the powerful anti-angiogenic thrombospondins (THBS1, THBS2, and THBS3), tenascin C, about 15 genes encoding for collagen and cysteine-rich protein 61. Stromal cells, which present a lack of their peculiar features, seem more like epithelial components after a process of mesenchymal-epithelial transition that may help HPV replication in reprogrammed cells [8]. Tumor-associated fibroblasts induce also the direct proliferation of keratinocytes, releasing a ligand of epidermal growth factor receptor (EGFR), namely heparin-binding EGF like growth factor (HBEGF), against which further studies may create monoclonal antibodies [8].

### 4.2. Synergic Effects of Epithelial High-risk HPV and Stromal ERα

Persistent infection by high-risk HPVs is crucial but needs another ally to induce tumor development, namely, estrogens that increase during certain conditions like pregnancy or for iatrogenic reasons (oral contraceptives’ assumption) [8]. Many epidemiological evaluations underline that woman affected by breast cancer in treatment with aromatase inhibitors were at reduced risk for CCx and its precursors thanks to lower E2 levels [9]. 

Since increased levels of E2 within the CCx stroma facilitate tumoral progression, a synergic effect between estrogens’ pathway and HPV-oncoproteins has been discovered. Thence, a recent study on transgenic HPV-transgenic mice evidenced the expression of a peculiar chemokine/cytokine panel in the presence of exogenous treatment even in the case of epithelial knock-down for ERα [9]. Notably, the synergic effect of E6/E7 and E2 accounts for 40% of up-regulated genes and 22% of the down-regulated genes [8]. In details, the proliferative/pro-angiogenic/pro-inflammatory signature of the cancer microenvironment is sustained by HPV alone but also in concert with the E2 cascade, mostly mediated by the stromal ERα enhanced by the presence of tumor-associated fibroblasts [8,9]. Therefore, the stromal estrogen pathway feeds a vicious cycle led by the HPV oncoproteins, expressed by epithelium in a mutual paracrine cross-talk. Recent evidence revealed that E6/E7 keratinocytes treated with estrogens (E6/E7+E2) carry pro-inflammatory and tumor-promoting genome panels. Among the highly expressed genes, it has been remarkably described the role of chemokines belonging to the ELR+ CXC family that bind CXCR2, namely CXCL1, CXCL2, CXCL3, and CXCL5. Interestingly, they join the pathway of tumor initiation and angiogenesis within the precancerous lesions and the further malignant step. Other up-regulated molecules within the E6/E7+E2 stroma are IL1A and IL1B, fibroblast growth factor 9 (FGF9) and HBEGF, which can cooperate in providing an inflammatory *milieu* and anti-apoptotic escape for HPV^+^ cells. According to Spurgeon et al. [8], the association between estrogen activity and HPV oncoproteins may orchestrate the mesenchymal concentration of pro-inflammatory and pro-angiogenic chemokine/cytokine, tumor-growth signals, cell-to-cell adhesion molecules, apoptosis escape, mesenchymal-to-epithelial transition and ECM plasticity in a transgenic mice model (E2+E6/E7) [8]. Since the epithelial receptor for E2 is not critical for CCx biological progression as the stromal one, blocking the mesenchymal expression of ERα in mice with E7^+^ keratinocytes determines the regression of cervical intraepithelial lesions [9]. Indeed, anti-estrogens down-regulate the amount of pro-inflammatory/pro-angiogenic molecules and growth factors (such as HBEGF) secreted by tumor-associated fibroblasts [8]. In the near future, the crucial role of stromal ERα in the cancerous promotion process of high-risk HPV^+^ epithelium could become a potential target for therapeutic purposes.

**Figure 4:**
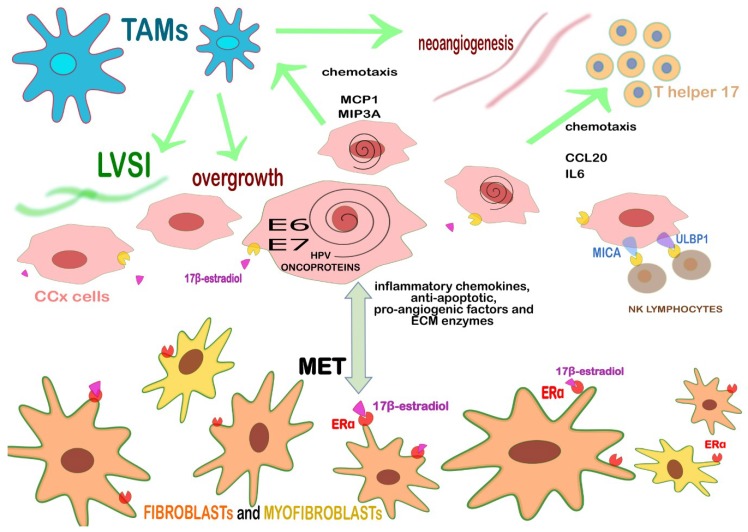
Crosstalk between CCx’s cells and the surrounding stroma. The peritumoral stromal cells in CCx is mainly composed by ERα+ fibroblasts and myofibroblasts, TAMs and TILs. Fibroblasts and myofibroblasts, activated by the binding 17-β-estradiol and its stromal receptor ERα secrete inflammatory chemokines, anti-apoptotic, pro-angiogenic factors and ECM enzymes. They also go through the process of Mesenchymal-epithelial transition (MET). Persistently high-risk HPV+ keratinocytes that became CCx cells exert a chemoattraction for monocytes (MCP1 and MIP3A), Th17 lymphocytes (CCL20, IL6) but also NK cells in advanced stages. The proliferative/pro-angiogenic/pro-inflammatory signature of the TME is sustained by the oncoproteins (E6 and E7) of high-risk HPV but also in concert with the E2 cascade, mostly mediated by the stromal ERα enhanced by the presence of tumor-associated fibroblasts. MICA/B and ULBP1 expressed by CCx cells bind NKG2D, a receptor located on the NK lymphocyte’s surface. Th17 lymphocytes play a role in sustain a chronic pro-inflammatory/pro-tumoral effect within TME, whereas NK lymphocytes increase the prognosis thanks to their cytotoxic activity against CCx cells. Other important actors within TEM are CD163^+^ M2 TAMs because they promote angiogenesis, LVSI, lymph node metastasis, and tumor overgrowth. The green arrows stand for positive *stimuli*.

An overall view of the main cell types of the CCx’s TEM and their principal features are reported in Table 3 and in Figure 4 below.

## 5. The Role of TAMs in Endometrial and Cervical Cancer and Their Translational Relevance in Gynecological Malignancies

### 5.1. TAMs in EC and CCx

With respect to cervical and endometrial cancer, TAMs have been found to correlate with several unfavorable histological and clinical features [82]. Compared to benign tissues, endometrial and cervical carcinomas display higher CD68^+^ and CD163^+^ macrophages density [43,44,45]. Specifically, CD163^+^ M2-macrophages positively correlate with microvessel density, angiogenesis, lymphovascular space invasion, lymph node metastasis, as well as with higher FIGO stage, higher histological grade, increased expression of Ki-67 and p53 and a worse PFS [83,84,85,86]. In endometrial adenocarcinoma, TAMs inversely correlate with the expression of progesterone receptor [76] and with the enhanced sensitivity and addiction of endometrial cancer cells for estradiol [75]. In cervical cancer, a significant increase in VEGF-C expressing TAMs is correlated with tumor lympho-angiogenesis and lymphatic spread [86]. Moreover, in cervical adenocarcinoma patients, the presence of PD-L1^+^ TAMs and a lower M1/M2 ratio are associated with poor disease-specific survival [87,88]. 

### 5.2. Translational Significance of TAMs in Gynecological Malignancies

The ongoing identification of the multifaceted function of TAMs is providing the opportunity for the development of novel therapies for gynecological cancers [82]. Furthermore, the affinity of TAMs to the peritoneal TME and the ascites offers future potential for targeted intraperitoneal treatments, in combination with classical chemotherapy drugs [82]. 

The main therapeutic strategies include the following:

(i) re-education of TAM function toward an antitumor, immunostimulatory function [40]. With this respect, the first-line treatment with Paclitaxel (PCX) has been proven useful to reprogram TAMs into a pro-inflammatory M1-profile via TLR4 signaling and this might contribute, at least in part, to the antitumor effect of PCX [89]. In HGSC, neferine (an alkaloid from lotus seed embryo) inhibits M2-macrophage polarization via mTOR/p70S6K pathway impairment, leading to an anti-angiogenetic effect [90]. Plant-derived products, such as 9-hydroxycanthin-6-one and deoxyschizandrin, have been found to inhibit M2-macrophages polarization in ovarian cancer [91,92]. Since the diverse function of macrophages are underpinned and sustained by specific metabolic signatures, targeting immuno-metabolism by the selective inhibition of the specific enzymes rather than ablation of general macrophage function is destined to represent a new attractive strategy to revert their function from M2 to M1 like [69,70]; 

(ii) blocking monocytes migration to the TME. Since EOC cells release the chemokine CCL2 to attract monocytes and convert them to TAMs within the TME [93], targeting CCL2 with selective antibodies might reduce monocyte infiltration and TAM differentiation. However, a clinical trial using an anti-CCL2 antibody, known as Carlumab or CNTO 888, has been completed (NCT01204996) with no significant results on tumor responses [45]. Bisphosphonates (such as clodronate, alendronic acid) are able to potentiate adoptive immunotherapy by depleting monocytes/macrophages in ovarian cancer [94]. Several inhibitors of the CSF/CSF-1R pathway, involved in the differentiation of monocytes into macrophages, both small molecules and antibodies, have been developed and are being studied in clinical trials. For example GW2580, a selective CSF1R kinase inhibitor, significantly reduces ascites fluid buildup, decreases the number of infiltrating TAMs and also repolarizes M2 macrophages within the tumor microenvironment to an M1-like phenotype [95];

(iii) activation of phagocytosis activity by macrophages. This strategy is being exploited thanks to the antigen CD47, expressed by ovarian cancer cells, that functions as a “don’t eat me signal” through ligation of signal-regulatory protein alpha (SIRPα) expressed on macrophages [96]. Targeting CD47 with antibodies induces a switch of TAMs polarization toward the anti-tumoral function by enhancing TAM mediated phagocytosis of cancer cells [96]. Clinical trials targeting CD47 are underway both as a single agent and in combination with anti-EGFR cetuximab [82];

(iv) blockade of PD-L1 on macrophages to increase activation of T cells. The robust expression of PD-L1 on TAMs likely contributes to T cell exhaustion and tumor-mediated immunosuppression. Several antibodies such as pembrolizumab, nivolumab and avelumab, targeting the PD-1/PD-L1 axis, are able to promote survival, activation and proliferation of T cells and are being tested clinically in patients with recurrent or refractory, platinum- and taxane-resistant ovarian cancer [97,98];

These novel strategies are partly showing their beneficial effects in ovarian cancer. However, more research is needed to achieve clinical efficacy and to evaluate the effects of existing and already used drugs, including trabectedin (an agent that intercalates with the DNA and causes DNA damage in tumor cells), bevacizumab (monoclonal antibody directed against VEGF) or olaparib, inhibitor of poly ADP-ribose polymerase (PARP), an enzyme involved in DNA repair.

## 6. Conclusions

The main properties of malignant tumors’ invasion, progression, and subsequent dissemination rely on cancer cells and the reaction of TME to their action. Considering the most common gynecological malignancies, intense crosstalk between the tumor and the surrounding stroma orchestrates the cancer behavior even in the gynecological field.

The translational significance of the tumor-associated stromal cells should be considered in developing new target therapy against instructed CAFs and activated pericytes since they strongly influence the TME, especially in the EOC. 

Recent studies on immunotherapy have developed drugs able to restore the anti-tumorigenic balance of TILs, mainly cytotoxic T cells, T-regs and TAMs. 

Estrogens and their stromal receptor (ERα) in EC and CCx orchestrate the dialogue between the cancer cells and the mesenchymal micro-environment offering interesting translational results.

The action of E2 is powered by persistent high-risk HPV cervical infection, even though screening and vaccine campaigns have been decreasing the HPV-incidence since their introduction in developed Countries.

## Figures and Tables

**Table 1:** Main cell types of the Epithelial Ovarian Cancer’s (EOC) TEM, their principal role in EOC pathogenesis and the possible translational value.

Cell Type	Pathogenetic Role	Translational Possibilities and Hypothesis
CAFs	Recruited by PDGFβ and activated by TGFβ/VCAN, CXCL.	Inhibiting PDGF- β signaling.
	They promote EOC cells’ motility, overgrowth, neo-angiogenesis, and invasion.	Inhibiting pro-angiogenic factors VEGF, FGF-2.
α-SMA^+^ pericytes	Recruited by the PDGFβ, their rate and genetic signature correlate with proliferation, migration and cell motility of EOC. They reduce cell-to-cell adhesion without affecting angiogenesis significantly. They build a structural guide for the cancer new vessels.	Inhibiting of PDGF-β signaling.
CD103^+^ NK lymphocytes	Tumor growth restriction, innate immunity. Activated by NKG2DLs.	Neutral competitor ligand for NKG2D to prevent the NK cells’ anergy.
	High levels of circulating ULBP2 (NKG2DL) fragments could down-regulate the EOC’ expression of NKG2D	Inhibitors of ULBP2 to prevent the impairment of the NK cells’ cytotoxic activity
T helper 1 lymphocytes	Tumor growth restriction mediated by CD8^+^ activation. Diapedesis and differentiation promoted by INFγ, IL-2, and lymphocyte-attracting chemokines. They secrete in turn interferon γ (INF γ) and IL-2 to chemoattract cytotoxic TILs.	
	Diapedesis inhibited by endothelin and VEGF.	Possible chemoattraction and diapedesis within TEM via inhibition of endothelin and VEGF
T helper 17 lymphocytes	Pro-inflammatory TILs that stimulate CD8^+^ activation.	
	Diapedesis inhibited by endothelin and VEGF.	Possible chemoattraction and diapedesis within TEM via inhibition of endothelin and VEGF.
CD8^+^ CD103^+^ (CD137^+^) cytotoxic T lymphocytes	Tumor growth restriction, acquired immunity, cytotoxic activity after interaction with T helper. Presence in serous > endometrioid > clear cell > mucinous histological subtype. Diapedesis promoted by INFγ, IL-2, and lymphocyte-attracting chemokines.	Contemporary inhibition of CTLA-4 and PD-1, associated with vaccination. Agonistic antibodies specific for CD137 alone or in association with inhibition TIM3. Autologous CD8^+^ TILs cultured with IL2, expanded and then infused. Chimeric T cell receptor restricted for HLA-A2 that can bind a specific epitope of HER2.
	Diapedesis inhibited by endothelin and VEGF.	Possible chemoattraction and diapedesis within TEM via inhibition of endothelin and VEGF.
CD20^+^ B lymphocytes	Tumor growth restriction. They increase the survival rate of CD8^+^ TILs. CD20^+^ TILs might act as APCs (positivity for MHC I/II, CD80, CD86, and CD40) → antigen *reservoir* preventing CD8^+^ anergy form persistent stimulation due to tumor lysis activity.	
	Diapedesis inhibited by endothelin and VEGF.	Possible chemoattraction and diapedesis within TEM via inhibition of endothelin and VEGF.
CD4^+^CD25^+^ FOXP3^+^T_regs_	They inhibit the cytotoxic functions of TILs releasing inhibitory cytokines (TGF-β and IL-10) or via a direct cell-to-cell block.	T_regs_ depletion or shift into T helper 17 (IL2).
	Activated by CCL28 under hypoxia condition and in the presence of B7H4^+^ TAMs.	Inhibition of CCL28; immunotherapy against TAMs.
TAMs	CCL2/CCR recruited. TAMs promote immunosuppressive activity: T_regs_ trafficking to TEM (CCL22) and inhibit T-cell cytotoxicity (B7-H4, PD-L1).	Reducing monocytes chemoattraction within TEM (bisphosphonates, inhibitors CCL2 antibodies). Inhibiting the PD-L1/2 checkpoint to reactivate cytotoxic T cells. Inhibition of PD-1/PD-L1 axis using target antibodies (pembrolizumab, nivolumab, avelumab) to promote survival, activation, and proliferation of cytotoxic T cells.
	TAM offer metabolic support for EOC cells (glutamine).	Depleting extracellular glutamine.
	Activated by IL10, IL6, TGFβ, PUFAs acquiring an M2-like polarization state. TAM promote angiogenesis (VEGF).	Shifting the M2 to M1-like (pro-inflammatory and anti-angiogenetic) via TLR4 signaling (PCX), via inhibition of mTOR/p70S6K (neferine), via the inhibition of inhibitors of the CSF/CSF-1R pathway (GW2580, a selective CSF1R kinase inhibitor) or using 9-hydroxycanthin-6-one, deoxyschizandrin).
	TAMs promote metastasis dissemination thanks to the secretion of CCL18 and matrix support and growth factors (EGF) within EOC spheroids floating in the peritoneal fluid bound together by integrins and ICAM-1 via CD11b/c binding.	
		Re-activation of phagocytosis inhibiting CD47 (EOC cells “don’t eat me signal” that binds TAMs’ SIRPα).

**Table 2:** Main cell types of the EC’s TEM, their principal role in endometrial cancer (EC) pathogenesis and the possible translational value.

Cell Type	Pathogenetic Role	Translational Possibilities
ERα^+^ fibroblasts and myofibroblasts	Juxtacrine and paracrine action on endometrium with the secretion of anti-apoptotic and proliferative factors. Loss of β-catenin and EMT-associated proteins (TWIST, SNAIL-SLUG) in an opposite subset of EC cells → enable the near cancer cells to migrate, invade and to escape from apoptosis.	Targeting stromal ERα or the further cascade-molecules: IGF1, TGF and cell-cycle-related proteins, such as MAD2L1, CDKN1A, and CEBPβ.Targeting stromal ERα might revert also the multistep tumoral process since it is influenced by estrogens.
CD163^+^ M2 TAMs	Promote angiogenesis, LVSI, lymph node metastasis, tumor overgrowth.	Re-education toward an antitumor, immunostimulatory function (PCX); blocking monocytes migration to the TME; activate the phagocytic activity of TAMs; blockade of PD-L1 on TAMs (avelumab, nivolumab pembrolizumab).

**Table 3:** Main cell types of the CCx’s TEM, their principal role in CCx pathogenesis and the possible translational value.

Cell-Type	Pathogenetic Role	Translational Possibilities
Persistently high-risk HPV+ keratinocytes	Inhibition of inflammation in early stages; progressive chemoattraction for monocytes (MCP1 and MIP3A), Th17 (CCL20, IL6) but also NK cells in advanced stages	Targeting EGFR, CCL2 (also known as MCP1); CCL20, (also known as MIP3A); IL6
ERα^+^ fibroblasts and myofibroblasts	Mesenchymal-epithelial transition Secretion of inflammatory chemokines, anti-apoptotic, pro-angiogenic factors and ECM enzymes	Targeting stromal ERα, IL1A and IL1B, FGF9, HBEGF, CXCR2 and its ligands CXCLs (mainly CXCL5 and CXCL1), MMP9.
CD163^+^ M2 TAMs	Promote angiogenesis, LVSI, lymph node metastasis, tumor overgrowth	Re-education toward an antitumor, immunostimulatory function (PCX); blocking monocytes migration to the TME; activate the phagocytic activity of TAMs; blockade of PD-L1 on TAMs (avelumab, nivolumab pembrolizumab)
Th_17_ lymphocytes	Chronic pro-inflammatory/pro-tumoral effect	Targeting CCL20 and IL6; re-education under IL2 stimuli
NK lymphocytes	Innate immune activity against tumoral cells expressing MICA and ULBP1 (NKG2DLs)	Clonal autologous expansion; vaccines against MICA and ULBP1

## Abbreviations

EOC	Epithelial ovarian cancer
BM-MSCs	Bone-Marrow-Derived Mesenchymal Stem Cells
AOCS	Australian Ovarian Cancer Study
TME	Tumor Micro-Environment
EMT	Epithelial-Mesenchymal Transition
ECM	Extra-Cellular Matrix
CCL	Chemokine, C-C motif, Ligand
Th17	T-helper 17
HPV	Human Papilloma Virus
α-SMA	Alpha Smooth Muscle Actin
PDGFβR	Platelet Derived Growth Factor Beta Receptor
CAFs	Cancer-Associated Fibroblasts
FAP	Fibroblast Activation Protein
MCAM/CD146	Melanoma Cell Adhesion Molecule/ Cluster of Differentiation 146
CD	Cluster of Differentiation
FGF/FGFR	Fibroblast Growth Factor/ Fibroblast Growth Factor Receptor
VCAN	VersiCAN gene
VEGF-A	Vascular Endothelial Growth Factor-A
CXCL	Chemokine C-X-C motif ligand
PFS	Progression Free Survival
OS	Overall Survival
LCA	Leukocyte Common Antigen
TILs	Tumor-Infiltrating Lymphocytes
T_regs_	T-regulatory cells
NK	Natural Killer
HSGC	High Serous Grade Carcinoma
TRM	Tissue Resident Memory
HLA	Human Leucocyte Antigens
IL	InterLeukin
INF γ	INterFeron γ
PD-L1	PD- Ligand 1
CTLA	Cytotoxic T-Lymphocyte-Associated Protein 4
MHC	Major Histocompatibility Complex
TP53	Tumor Protein 53
APCs	Antigen-Presenting-Cells
FOXP3	Fork Head Box 3
TGF-β	Tumor Grow Factor β
TAMs	Tumor-Associated Macrophages
VEGF	Vascular Endothelial Growth Factor
TNF	Tumor Necrosis Factor
PLCγ	PhosphoLipase Cγ
JAK/ STAT	JAnus Kinase/Signal Transducer and Activator of Transcription
PI3K	PhosphoInositide 3 Kinase
Tim-3	T-cell immunoglobulin and mucin-domain-containing-3
MIC A/B	Major histocompatibility complex class I-related chains (MIC) A and B
ULBPs	UL-16 binding proteins
NKG2D	Natural killer group 2, member D
EC	Endometrial Cancer
PTEN	Phosphatase and Tensin homolog
CTNNB1	Catenin Beta 1
PIK3CA	Phosphatidylinositol 3-kinase
MLH1	MutL Homolog 1
POLE	DNA POLymerase Epsilon
HNPCC	Hereditary Non-Polyposis Colon Cancer
ER α	Estrogen Receptor α
ESR1	(gene) EStrogen Receptor 1
E2	17β-estradiol
IGF1	Insulin-like Growth Factor 1
TGF	Tumor Growth Factor
MAD2L1	Mitotic Arrest Deficient 2 Like 1
CDKN1A	Cyclin-Dependent Kinase Inhibitor 1A
EH	Endometrial Hyperplasia
PR	Progesterone Receptor
CCx	Cervical cancer
pRB	Retinoblastoma protein
MCP1	Monocyte Chemoattractant Protein 1
MIP3A	Macrophage Inflammatory Protein-3α
HSIL	High-grade Squamous Intraepithelial Lesion
CCR	C-C motif Chemokine Receptor
LSIL	Low-grade Squamous Intraepithelial Lesion
C/EBPβ	CCAAT Enhancer Binding Protein Beta
MMP-9	Matrix-Metalloproteinase
FIGO	International Federation of Gynaecology and Obstetrics
THBS	Thrombospondins
EGFR	Epidermal growth factor receptor
HBEGF	Heparin Binding EGF Like Growth Factor
FGF9	Fibroblast Growth Factor 9
PUFAs	Polyunsaturated Fatty Acids
Evs	Extracellular Vesicles
VEGFR	Vascular Endothelial Growth Factor Receptor
EGF	Endothelial Growth Factor
EGFR	Endothelial Growth Factor Receptor
PCX	Paclitaxel
TLR	Toll-Like Receptor
SIRPα	Signal-Regulatory Protein Alpha
CSF-1	Colony-Stimulating Factor 1
CSF1-R	Colony-Stimulating Factor 1 Receptor
LVSI	LymphoVascular Space Invasion
MET	Mesenchymal-epithelial transition
